# Effects of Different Flotation Agents on the Nucleation and Growth of Potassium Chloride

**DOI:** 10.3390/molecules28237923

**Published:** 2023-12-04

**Authors:** Guangle Wang, Xiao Bian, Zeren Shang, Weibing Dong, Yi Zhang, Songgu Wu

**Affiliations:** 1 School of Chemistry and Chemical Engineering, Qinghai Minzu University, Xining 810007, China; guanglewang5018@tju.edu.cn (G.W.); bianxiao1036@163.com (X.B.); wbdong@tju.edu.cn (W.D.); 15373199837@163.com (Y.Z.); 2 State Key Laboratory of Chemical Engineering, School of Chemical Engineering and Technology, Tianjin University, Tianjin 300072, China; shangzeren@tju.edu.cn

**Keywords:** potassium chloride, flotation agent, nucleation kinetics, growth mechanism, crystallization

## Abstract

The flotation agent is an important collector in the production of potassium chloride and is brought into the crystallization stage with the reflux of the mother liquor. Octadecylamine Hydrochloride (ODA), 1-Dodecylamine Hydrochloride (DAH) and Sodium 1-dodecanesulfonate (SDS) were selected to study their effect on the nucleation of potassium chloride. Focused Beam Reflectance Measurement was used to collect the nucleation-induced periods of KCl in the presence of flotation agents at different supersaturations. Then, empirical equations, classical nucleation theory and growth mechanism equations were employed for data analysis. It was found that the presence of flotation agents increased the nucleation sequence *m*, and *m*(ODA) > *m*(SDS) > *m*(DAH) > *m*(H_2_O). In addition, the interfacial energy data obtained using classical nucleation theory suggest that the flotation agents used in our paper promoted the homogeneous nucleation of KCl (reduced from 5.3934 mJ·m^−2^ to 5.1434 mJ·m^−2^) and inhibited the heterogeneous nucleation of KCl (increased from 2.8054 mJ·m^−2^ to 3.6004 mJ·m^−2^). This investigation also revealed that the growth of potassium chloride was consistent with the 2D nucleation-mediated growth mechanism, and the addition of flotation agent did not change the growth mechanism of potassium chloride. Finally, the particle size distribution results were exactly consistent with the order of nucleation order *m*. The study of nucleation kinetics and growth mechanisms of different flotation agents on potassium chloride can provide guidance for optimizing the production process of potassium chloride and developing new flotation agents.

## 1. Introduction

**Figure 1:**
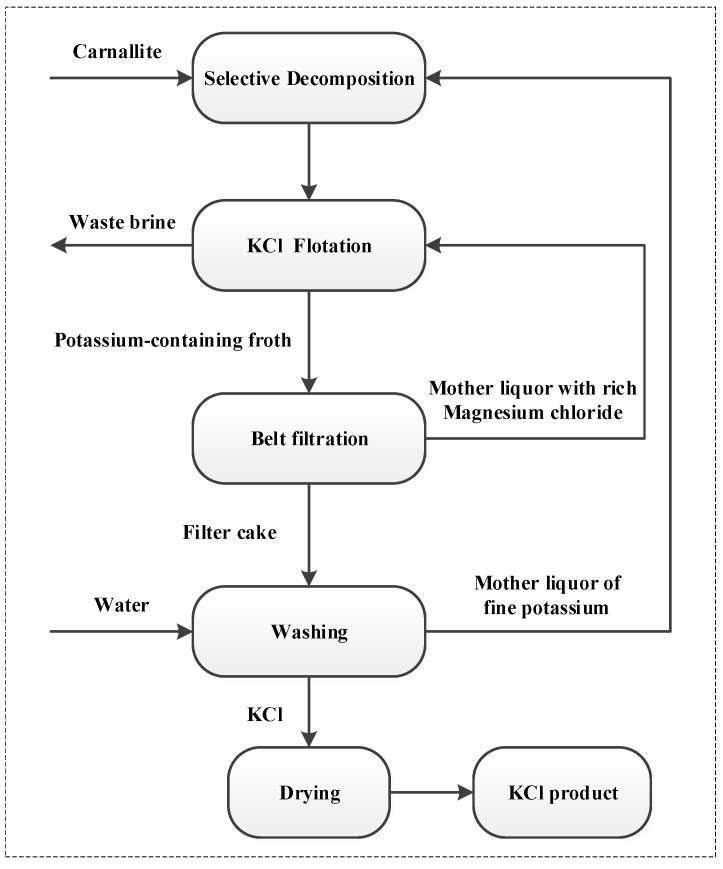
Process flow chart of cold decomposition–direct flotation technology of potassium chloride.

Potassium is one of the necessary nutrients for plant growth, and in agricultural production, potassium fertilizer is necessary in order to maintain high yields [1]. KCl is the most common form of potash and more than 80% is produced using flotation [2]. Carnallite obtained from salt lake brine is the main raw material of sylvite, which is produced by a combination process of selective decomposition, flotation, filtration and dehydration, as shown in Figure 1 [3,4]. 

The surfactant is a collector in the flotation process of potassium chloride and acts as an additive to the crystallization of potassium chloride with the partial return of the mother liquor to the selective decomposition stage. Although the amount of surfactant returned is relatively small, studies have shown that even small amounts of additives in the crystallization process may affect the nucleation and growth processes [5,6,7]. At present, there have been a large number of research studies on the kinetics of potassium chloride. Haneveld, H. B. K. described the growth and dissolution of KCl crystals in solution and found that the growth and dissolution rates depend on supersaturation [8]. Sarig, S. accurately determined the supersaturation of potassium chloride in a mixed-suspension mixed-product removal (MSMPR) crystallizer and correlated the nucleation rate and growth rate with supersaturation [9]. Linnikov, O. D. studied the kinetics of the spontaneous crystallization of potassium chloride from aqueous and aqueous ethanol solutions and determined the growth rate and activation energy of potassium chloride [10]. Li, X. studied the nucleation process and crystallization kinetics of potassium chloride, and the relationship between particle size and growth rate was correlated in a continuous MSMPR crystallizer [11]. Zheng, Y. studied the nucleation, growth and aggregation kinetics of potassium chloride using population balance equations and explored the functional relationship between particle size and crystal growth rate [12]. However, most of them focused on the bulk nucleation kinetics or the growth process and growth mechanism of potassium chloride. To our knowledge, few researchers pay attention to the investigation of the primary nucleation of potassium chloride in the presence of flotation agents. As the initial stage of the crystallization process, nucleation directly affects the polymorph, purity, particle size, particle size distribution and other properties of crystal products, as well as the drying and transportation of subsequent products [13,14,15,16]. Therefore, three surfactants (ODA, DAH, SDS) commonly used for potassium chloride flotation were selected in this paper to study their effects on the nucleation behavior of potassium chloride, including both cationic and anionic surfactants, and the length of the hydrophobic carbon chain were also considered [17,18,19]. The study of the effect of different flotation agents on the nucleation behavior of KCl can provide guidance for the optimization of KCl production processes and the development of new flotation agents from the perspective of crystallization.

## 2. Results and Discussion

### 2.1. Solubility of KCl in the Presence of Flotation Agents

The solubility of KCl in aqueous solution with different flotation agent contents was measured at a temperature of 298.15 K. The solubility is expressed by mass fraction w (due to the small mass proportion of the flotation agents, the quality of the flotation agents was ignored in the calculation process):

(1) $$w=\frac{m_{2}-m_{0}}{m_{1}-m_{2}}$$

where $m_{0}$ represents the mass of the beaker, $m_{1}$ refers to the mass of the beaker and solution and $m_{2}$ is the mass of beaker and solute. 

**Figure 2:**
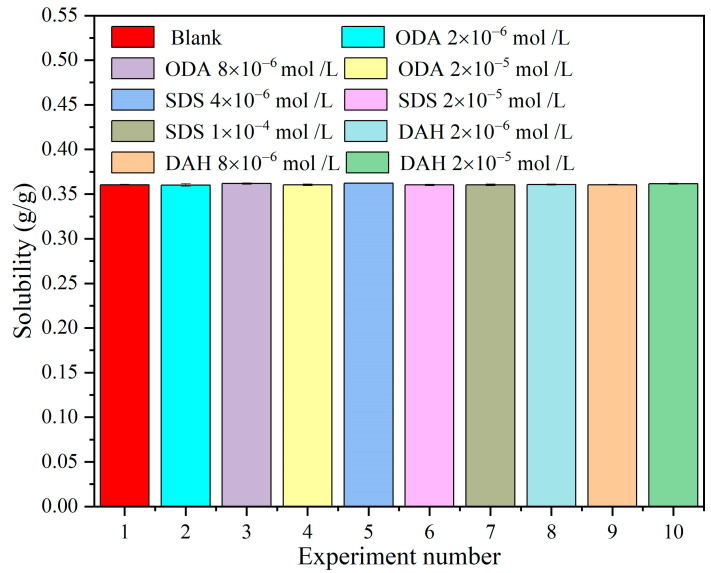
The effects of different flotation agent concentrations on the solubility of KCl at 298.15 K.

The solubilities of KCl in the presence of flotation agents are shown in Figure 2. The graph shows that ODA, SDS and DAH have little effects on the solubility of potassium chloride within the selected concentration range. According to the solubility, KCl solutions with corresponding supersaturations were able to be accurately prepared during the whole experiment.

### 2.2. Determination of Induction Period of Potassium Chloride in the Presence of Flotation Agents

**Figure 3:**
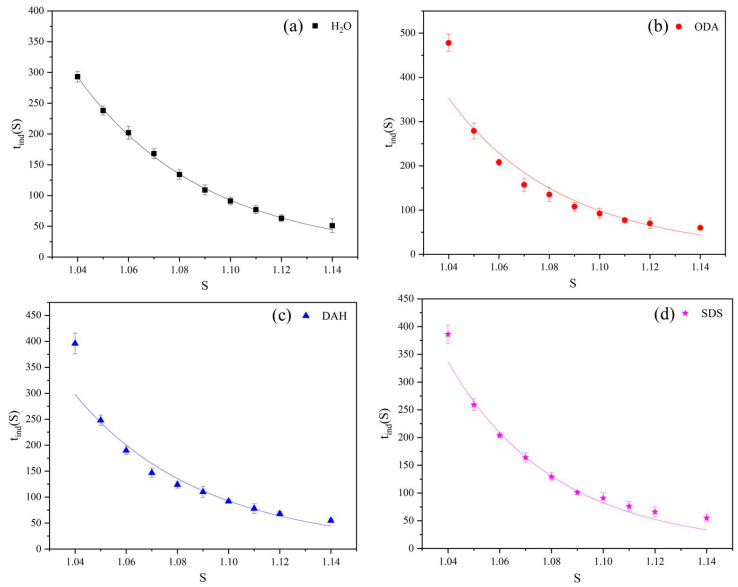
The relationship between the induction periods of potassium chloride and supersaturation ratios in the presence of different flotation agents: (**a**) H_2_O, (**b**) ODA, (**c**) DAH, (**d**) SDS.

The induction period experiments of potassium chloride were carried out at the temperature of 298.15 K, the concentrations of ODA, DAH and SDS were all selected at 2 × 10^−5^ mol/L and the supersaturation ratios of potassium chloride were 1.04, 1.05, 1.06, 1.07, 1.08, 1.09, 1.1, 1.11, 1.12 and 1.14, respectively. The results are shown in Figure 3.

In general, the following empirical equation can be used to describe the relationship between induction period and supersaturation ratio:

(2) $$t_{ind}=\frac{K_{1}}{S^{\delta }}$$

where $K_{1}$ and δ are empirical constants. The induction periods of potassium chloride in the presence of different flotation agents are related to supersaturation ratios, and the empirical constants $K_{1}$ and δ were obtained from Formula (2), as is shown in Table 1.

From Figure 3 and Table 1, we know that in the presence of different flotation agents, the induction periods and supersaturation ratios can be well correlated by Equation (2). At the same time, Figure 3 leads us to the conclusion that the induction periods decrease with the increase in supersaturation ratio, indicating that the nucleation rate increases, and the presence of flotation agents did not change the trend. Nucleation in solution is the result of the formation of clusters when the movement and collision of solute molecules reach a critical size. The supersaturation ratio can promote the movement and collision of solute molecules, increase the nucleation rate and reduce the induction period. This phenomenon is consistent with classical nucleation theory.

**Figure 4:**
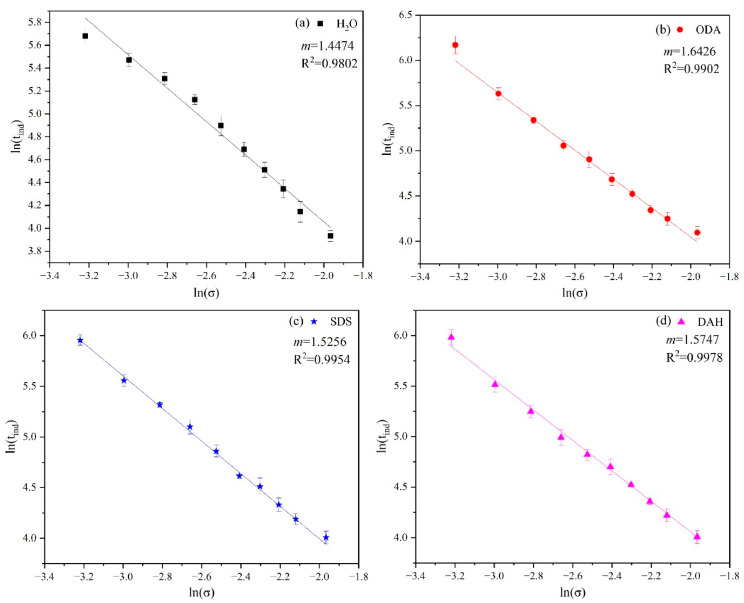
Plot of $lnt_{ind}$ and $ln\left(\sigma \right)$ for potassium chloride crystallization in the presence of different flotation agents: (**a**) H_2_O, (**b**) ODA, (**c**) SDS, (**d**) DAH.

According to Equation (6), we can obtain *m* by plotting $lnt_{ind}$ with $ln\left(\sigma \right)$ and fitting a line, and the results are shown in Figure 4. The *m* value of the nucleation order in pure water is equal to 1.4474, which is close to the reported result in the literature (*m* = 1.464) [20].

There is a good linear relationship between $lnt_{ind}$ and $ln\left(\sigma \right)$, and the presence of flotation agents leads to the increase in the nucleation order *m*. As is seen from Equation (3), under the same driving force, the larger the nucleation order, the larger the nucleation rate, resulting in a smaller product particle size. The order of the influence of the three flotation agents on the nucleation order *m* was ODA > SDS > DAH > H_2_O.

### 2.3. Nucleation Kinetics of Potassium Chloride in the Presence of Different Flotation Agents

**Figure 5:**
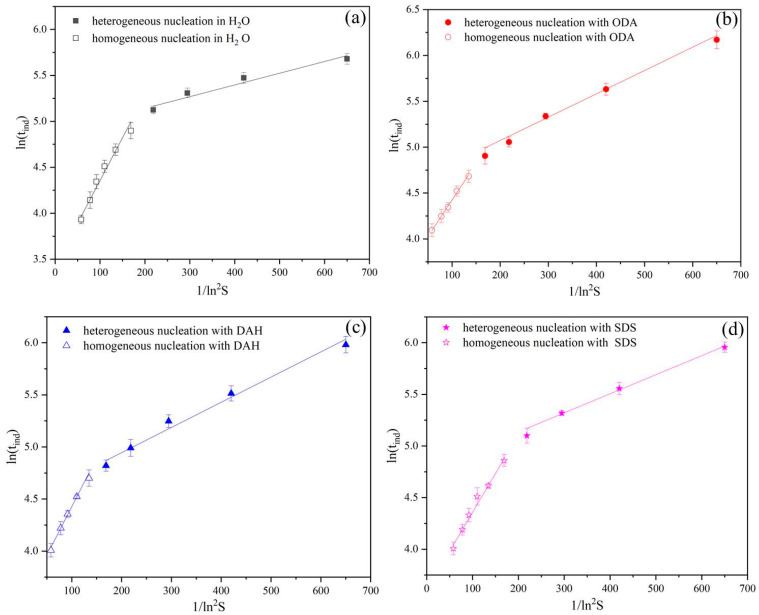
The relationship between ${lnt}_{ind}$ and ${ln}^{2}S$ in the potassium chloride induction period experiment in the presence of different flotation agents: (**a**) H_2_O, (**b**) ODA, (**c**) DAH, (**d**) SDS.

The correlation between induction period and supersaturation ratio for the primary nucleation process is evident from Equation (10). In order to better understand the nucleation behavior of KCl in the presence of different flotation agents, ${lnt}_{ind}$ was used to plot against ${ln}^{2}S$, and the results are shown in Figure 5. 

As is shown in Figure 5, the induction periods measured by the experiments in the presence of different flotation agents conform to the linear relationship of Equation (10), which consists of two straight lines with different slopes. The changes in the slopes indicate two different nucleation mechanisms and the lines with higher slopes indicate that homogeneous nucleation occurs under higher supersaturation ratios, while the line with a low slope indicates that heterogeneous nucleation occurs at a lower supersaturation ratio [21]. Therefore, a single linear relationship cannot be derived from the classical nucleation theory. Heterogeneous nucleation occurs on surfaces other than those of perfect crystals, such as crystallizer walls, impellers or dust particles that are always present during any crystallization process. These nucleation centers reduce the free energy barrier of nucleation and become the preferred sites for nucleation, which promotes heterogeneous nucleation. Therefore, homogeneous nucleation takes a long time to occur if in a low supersaturated solution. Homogeneous nucleation is dominant at a high supersaturation ratio, while heterogeneous nucleation mainly occurs at low supersaturation ratio.

Table 2 presents the results of the slopes and the corresponding interfacial energies of the two straight lines of homogeneous and heterogeneous nucleation, which can be obtained from Equation (10). The interfacial energy is an important thermodynamic parameter to characterize the crystallization ability of solute from solution and represents the difficulty of generating new phase. As is shown in Table 2, the estimated interfacial energies range from 2.8054 mJ·m^−2^ to 5.1604 mJ·m^−2^, which are of the same order of magnitude with other similar systems (NaCl 3.8 mJ·m^−2^, Na_2_SO_4_·10H_2_O 2.1~3.9 mJ m^−2^) [22,23]. The lower the interfacial energy, the more easily the solute crystallizes from the solution. The interfacial energies of heterogeneous nucleation are lower than that of homogeneous nucleation, which is consistent with our expectation. 

For homogeneous nucleation, flotation agents reduce the interfacial energies of the nucleation of KCl. SDS has the greatest effect on the homogeneous nucleation of potassium chloride, followed by DAH, and ODA has the least effect. For heterogeneous nucleation, the presence of flotation agents makes the interfacial energies increase, and heterogeneous nucleation becomes difficult. ODA has the greatest impact on heterogeneous nucleation, followed by DAH, and SDS has the least effect. The reason for the above results may be that the addition of flotation agents leads to a decrease in interfacial tension of the solution. At high supersaturation, the interfacial tension of the solution is reduced, resulting in the agglomeration of potassium chloride molecules, and the critical nucleation size is exceeded more easily. However, under low supersaturation, the interfacial tension decreases, leading to the weak priority position of the heterogeneous interface and difficult nucleation, so the interfacial energies increase. At the same time, according to the value of the intercept in Equation (10), we can also obtain the value of $J_{n}/K$, as K is the minimum detectable number density of accumulated crystals (m^−3^), which is a fixed but unknown value [24,25]. The trend of variations in $J_{n}$ in the presence of different flotation agents can be determined. From Table 2, we know that the influence of flotation agents on $J_{n}$ is the same as that on *γ*, and similar trends was also obtained in other research [26,27]. The lower values of $J_{n}$ may be due to a lower concentration of active sites or a lower value of attachment frequency.

The critical nucleation radius can be obtained using Equation (11). The results are shown in Table 3. The critical nucleation radius ranges from 6.48 to 41.62 Å, which is the same order of magnitude with other model substances (8 to 25 Å) [28]. The critical nucleation sizes of heterogeneous nucleation are smaller than that of homogeneous nucleation. Flotation agents reduce the critical sizes of homogeneous nucleation thus promote the nucleation. SDS has the most obvious promoting effect. For heterogeneous nucleation, the flotation agents increased the critical nucleation sizes of potassium chloride and inhibited nucleation, and the inhibitory effect of ODA was the strongest.

### 2.4. Growth Mechanism of Potassium Chloride in the Presence of Different Flotation Agents

According to Equations (15) and (17), the experimental data of induction periods under the different flotation agent contents were analyzed to determine the growth mechanism of potassium chloride. Since the crystal habit of potassium chloride is square, the *β* value is equal to 3. Additionally, *ν* = 1/2 or 1, so *n* is 5/2 or 4. The expressions of *F_u_*(*S*) corresponding to different growth mechanisms of potassium chloride are shown in Table 4.

**Figure 6:**
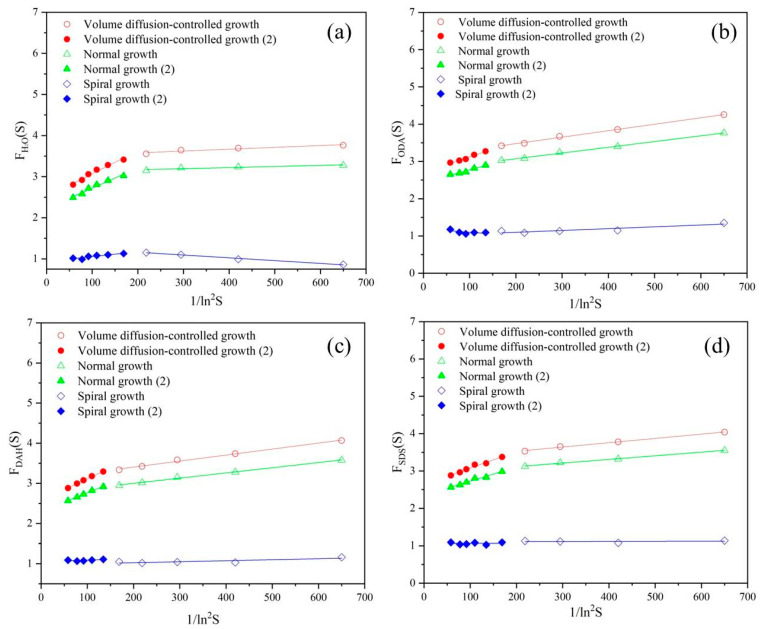
Dependence of *F_u_*(*S*) on *ln*^2^*S* under normal growth, spiral growth and diffusion-controlled growth mechanisms in the presence of different flotation agents: (**a**) H_2_O, (**b**) ODA, (**c**) DAH, (**d**) SDS.

**Figure 7:**
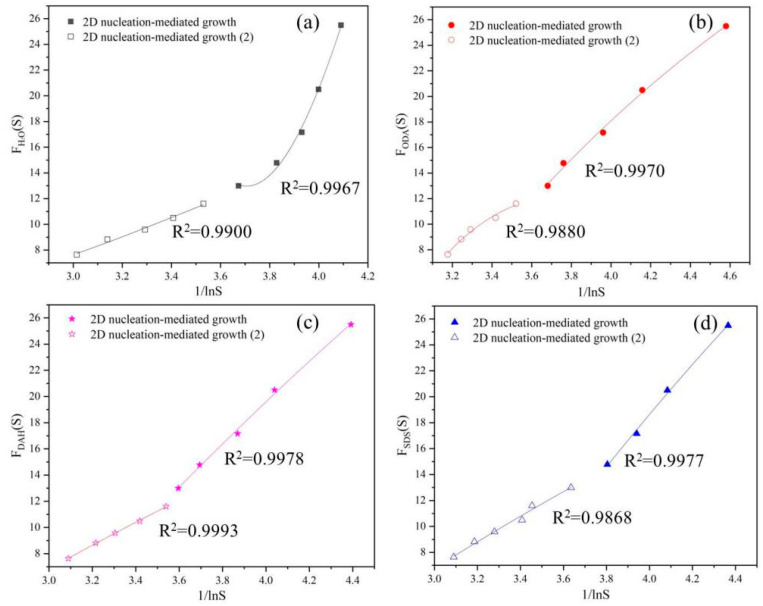
Dependence of *F_u_*(*S*) on *lnS* under 2D nucleation-mediated growth mechanism in the presence of different flotation agent: (**a**) H_2_O, (**b**) ODA, (**c**) DAH, (**d**) SDS.

The relationship between *F_u_*(*S*) and 1/*ln*^2^*S* was linear for normal growth, spiral growth and diffusion-controlled growth, while the relationship between *F_u_*(*S*) and 1/*lnS* was quadratic polynomial for 2D nucleation-mediated growth. The values of *F_u_*(*S*) were plotted with 1/*ln*^2^*S* or 1/*lnS* as is shown in Figure 6 and Figure 7, respectively. The quality of fitting was measured according to the values of R^2^ listed in Table 5 and Figure 7, and the growth mechanism of potassium chloride was judged using the results of fitting.

In Figure 6 and Figure 7, we performed segment processing when fitting lines and curves, instead of fitting in all supersaturation ratio ranges as in other previous studies [29]. It can also be seen from the figures that the effect of the segment fitting is a better. As can be seen from the Table 5, the fit degree of diffusion-controlled growth is higher when flotation agents were not added, and the fit degree of normal growth mechanism after flotation agents were added is also higher. However, comparing the R^2^ value in Figure 7, it is found that the growth of potassium chloride is in line with the 2D nucleation-mediated growth mechanism, and the addition of flotation agents did not change the growth mechanism of potassium chloride.

### 2.5. Influence of the Different Flotation Agents on the Crystal Habit and Particle Size Distribution of Potassium Chloride

**Figure 8:**
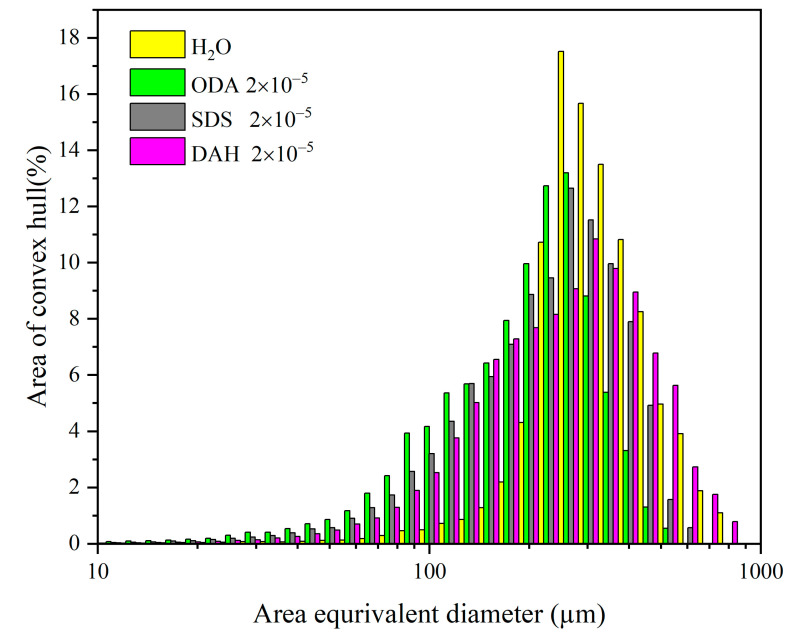
Particle size distribution of potassium chloride in the presence of different flotation agents (agitation rate 300 rpm, cooling rate 20 °C/h, supersaturation ratio 1.08).

**Figure 9:**
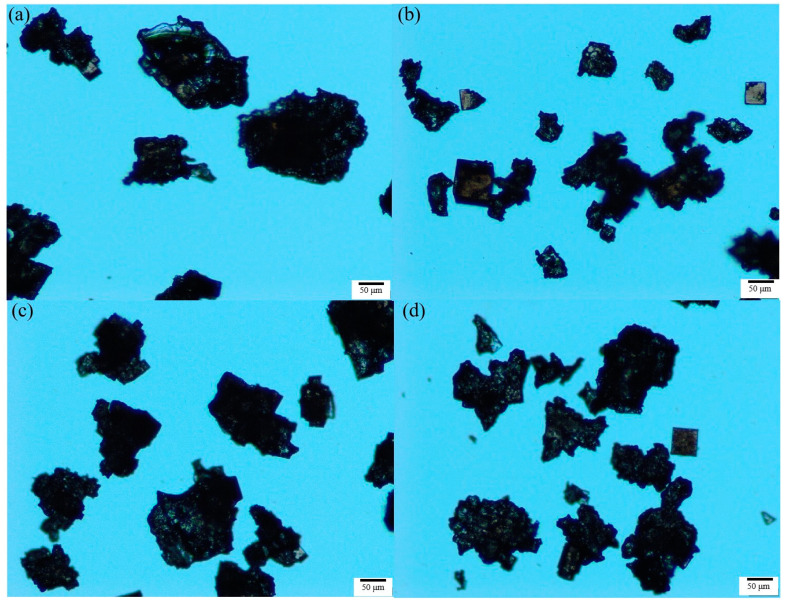
Crystal morphology of potassium chloride in the presence of different flotation agents: (**a**) H_2_O, (**b**) ODA, (**c**) SDS, (**d**) DAH (agitation rate 300 rpm, cooling rate 20 °C/h, supersaturation ratio 1.08, the scale in all figures is 50 µm).

Nucleation is a key step in the crystallization process that controls the crystal type, particle size distribution and other product qualities, and the nucleation process can be verified through the product quality. Therefore, the method described in Section 3.4 was used in this paper to investigate the effects of different flotation agents on the crystal habit and particle size distribution of potassium chloride. According to the preliminary experiment, the crystallization of potassium chloride was carried out at a cooling rate of 20 °C/h with a supersaturation ratio of 1.08, and the stirring rate was maintained at 300 rpm, with the flotation agent concentration fixed at 2 × 10^−5^ mol/L. The products were characterized via a polarizing microscope (Olympus-BX53, Olympus Corporation, Tokyo Metropolis, Japan) and particle size analyzer (Occhio 500nanoXY, Occhio s.a., Angleur, Belgium); the results are shown in Figure 8 and Figure 9. In order to understand the effect of flotation agents on the particle size of potassium chloride more directly, the particle size statistics of potassium chloride were obtained via a particle size analyzer, and the results are shown in Table 6.

From the results of particle size distribution in Figure 8 and Table 6, we can see that compared with cooling crystallization in pure water, the particle sizes of potassium chloride became smaller after adding flotation agents, and the particle size decreases in the order of DAH > SDS > ODA. Although the cumulative particle size distribution varies at D10 and D50, they do not affect the overall results of the particle size distribution. The results indicate that ODA had the greatest effect on the nucleation of potassium chloride under similar conditions of growth, agitation, coalescence and crushing, followed by SDS and DAH. 

Although the result of the interfacial energy data in Table 2 shows that the presence of flotation agents promoted homogeneous nucleation and inhibited heterogeneous nucleation, this could not be correlated with the results of particle size distribution. However, the results of the particle size distribution are exactly consistent with the size order of the nucleation order *m* obtained from the Figure 4, and the higher the nucleation order *m*, the higher the nucleation rate, resulting in a smaller product particle size. Combined with the data of nucleation order *m* and particle size distribution, it is suggested to reduce the circulation amount of fine potassium mother liquor in cold decomposition crystallization stage to obtain the potassium chloride products with larger particle size.

## 3. Materials and Methods

### 3.1. Materials

Potassium chloride (≥99.5%) was purchased from Tianjin Kaitong Chemical Reagent Co., Ltd. (Tianjin, China), DAH (98%) was purchased from Jiuding Chemical Technology Co., Ltd. (Chengdu, China), SDS (97%) was purchased from Tianjin Guangfu Fine Chemical Research Institute (Tianjin, China), and ODA (98%) was purchased from TCI (Shanghai, China) Chemical Trading Co., Ltd. (Shanghai, China). All chemicals were used without further purification. The deionized water used in the experiment was prepared via the ultra-pure water mechanism in the laboratory.

### 3.2. Measurement of the Solubility of KCl in the Presence of Different Flotation Agents

The solubilities of KCl in aqueous solution with different flotation agent contents were determined via the static method. Before the solubility measurement, flotation agent aqueous solutions of different concentrations were first prepared and placed for use. The selected flotation agent concentrations were all lower than the critical aggregation concentrations. For example, the critical aggregation concentration of ODA is 2.06 × 10^−5^ mol/L in saturated potassium chloride at 25 °C [30]. The concentrations of different flotation agents selected in the experiment are shown in Table 7. First, about 50 g of the above solution and excess KCl were added to a conical bottle, then it was plugged with a stopper and sealed with sealing film. Immediately, the conical bottle was placed in a thermostatic shaker (the uncertainty was 0.1 K), the shaker was kept shaking for 24 h at a constant temperature and then allowed to stand for 12 h. Afterwards, the supernatant was taken with a syringe and filtered using a filter membrane (0.45 µm). The liquid was infused into a pre-weighed mass (*m*_0_) and labeled vial. The total mass (*m*_1_) was weighed and heated on a heating plate at 60 °C. Meanwhile, we recorded the total mass (*m*_2_) of the vial after drying until the weight did not change. Samples were taken for 5 timepoints in each condition, and the average value of the 5 measurements was taken for the solubility data of KCl.

### 3.3. Determination of Induction Period of the KCl in the Presence of Different Flotation Agents

**Figure 10:**
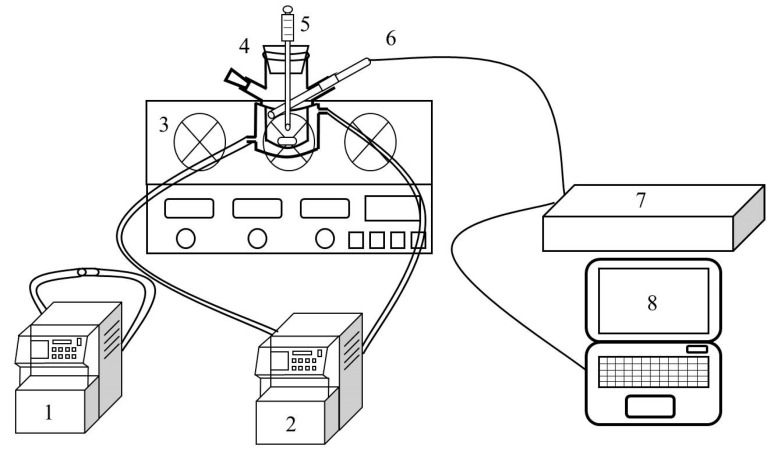
Experimental device of induction period determination (1: cooling circulating water machine (high temperature), 2: cooling circulating water machine (low temperature), 3: digital magnetic stirrer, 4: crystallizer, 5: electronic thermometer, 6: FBRM probe, 7: FBRM workstation, 8: computer).

The nucleation induction period of KCl was determined in a 100 mL jacket crystallizer with different flotation agent contents within the supersaturation ratio range from 1.04 to 1.14. Firstly, 50 g of aqueous solution containing the flotation agent was weighed into a jacket crystallizer, and a certain amount of potassium chloride was weighed according to the solubility data. Then, the supersaturated solution was placed on a magnetic agitator with the stirring rate kept at 300 rpm. Meanwhile, a cooling circulating water machine was used to maintain the crystallizer at 45 °C for 1 h to ensure the complete dissolution of KCl. After that, the crystallizer was quickly connected to another cooling circulating water machine with the temperature of 25 °C. When the temperature of KCl solution dropped to 25 °C, the timing began; the time until the first crystal appeared was the induction period. The experimental device is shown in Figure 10. 

**Figure 11:**
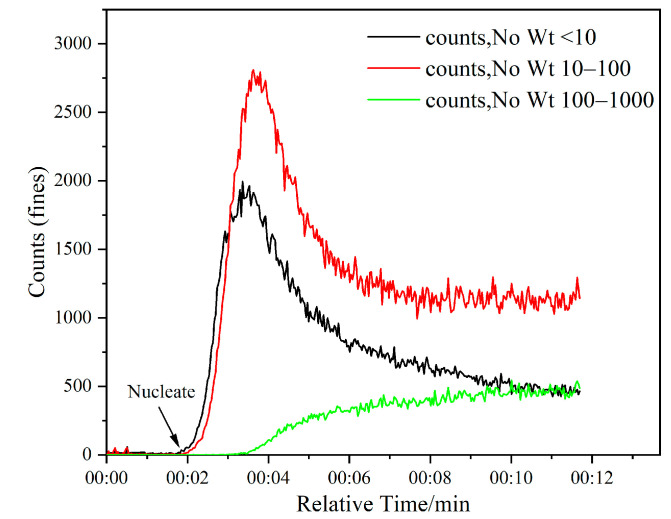
Monitoring the nucleation point of KCl using FBRM (DAH 2 × 10^−5^ mol/L, stirring rate 300 rpm, supersaturation 1.08).

The critical point of crystal emergence was detected via Focused Beam Reflectance Measurement (FBRM), and the nucleation point was when the counts increased, as is shown in Figure 11. The measurement of induction period was repeated 3 times in each experimental condition and the mean value was taken. The relative mean deviation was less than 5%.

### 3.4. Determination of the Crystal Habit and Particle Size Distribution of KCl

In order to study the effect of flotation agents on the crystallization behavior and the particle size distribution of KCl and to explore the nucleation rate indirectly through the particle size distribution data, the cooling crystallization method was adopted. First, 50 g solvent and potassium chloride corresponding to the supersaturation ratio were weighed and added to the jacket crystallizer, and the agitator was turned on (set at 300 rpm). Then the jacket crystallizer was connected to the cooling circulation water machine at 45 °C and maintained for 60 min to ensure that the solute was completely dissolved. Subsequently, we started cooling according to the set cooling rate; when the temperature of the solution dropped to the final temperature (25 °C), we stopped cooling and proceeded crystal cultivation for 1 h, then absorbed a small amount of crystal slurry under a microscope to observe the crystal habit and the aggregation of product. Then the crystal slurry was filtered with a vacuum pump, and the wet product was dried in an electric thermostatic drying oven at 50 °C for about 12 h. In the drying process, we turned the sample irregularly to prevent the product from caking. The particle size and particle size distribution were analyzed after the product completely dried.

## 4. Theory

### 4.1. The Nucleation Theory of Crystals

The rate of nucleation can also be considered as a function of concentration difference, so the rate of nucleation can be expressed as a power function:

(3) $$J=k∆c^{m}$$

where *m* is nucleation order, *k* is a nucleation coefficient, ${∆c=c-c}_{eq}$ stands for concentration difference, c is the initial concentration of potassium chloride and $c_{eq}$ is the equilibrium concentration of potassium chloride. The action of the concentration difference on nucleation rate was intuitively reflected by determining the size of *m*. If the effect of crystal growth on the induction period is not considered, then $t_{ind}=KJ^{-1}$, and combined with Equation (3), the relationship between induction period and concentration difference can be expressed as:

(4) $$t_{ind}=\frac{K}{k{\left(∆c\right)}^{m}}$$

In Equation (4), *K* is another nucleation rate coefficient. Because of the relative supersaturation $\sigma =∆c/c_{eq}$, we were able to obtain, by substituting Equation (4):

(5) $$t_{ind}=\frac{K}{k{\left(\sigma c_{eq}\right)}^{m}}$$

Taking the logarithm of both sides of Equation (5) produces:

(6) $$lnt_{ind}=ln\left[\frac{K}{k{\left(c_{eq}\right)}^{m}}\right]-mln\left(\sigma \right)$$

The nucleation order *m* at a given temperature was determined by the linear relationship between $lnt_{ind}$ and $ln\left(\sigma \right)$.

### 4.2. The Growth Theory of Crystals

In general, the primary nucleation rate and crystal growth rate together determine the induction period. Kashchiev et al. proposed a general expression for the induction period [31].

(7) $$t_{ind}=\frac{1}{JV}+{\left(\alpha /k_{v}JG^{n-1}\right)}^{1/n}$$

where V is the volume of solution, *α* is the volume fraction of the new phase, $k_{v}$ is the form factor, *G* is the growth rate and n=βv+1 (*β* = 1, 2, 3 growth dimensions, 0.5 < v < 1). This expression takes into account the two main mechanisms leading to the loss of metastability: the mononuclear mechanism (the first term on the right side of the equation) and the multinuclear mechanism (the second term on the right side of the equation). For large systems, we assumed that nucleation is due to a multinuclear mechanism, so the first term on the right side of the Equation (7) was ignored.

For three-dimensional nucleation, the steady state nucleation rate can be expressed as [32]:

(8) $$J=J_{n}exp\left(-B/{ln}^{2}S\right)$$

where $J_{n}$ is a dynamic factor and $S=c/c_{eq}$ is the supersaturation ratio, which is the ratio of solute concentration to saturation concentration. Combined with the definition of relative supersaturation, it is not difficult to conclude that σ=S−1. The thermodynamic parameter *B* is expressed as $B=\frac{k_{v}\gamma^{3}V_{m}^{2}}{k_{B}^{3}T^{3}}$; γ is the solid–liquid interface energy, which can be obtained from the thermodynamic parameter; $k_{B}$ is the Boltzmann constant; $V_{m}$ is the molar volume of KCl; and *T* is the Kelvin temperature.

Usually, the induction period is inversely proportional to the rate of nucleation. The induction period can be expressed as

(9) $$t_{ind}=\frac{K}{J_{n}}exp\left(\frac{B}{{ln}^{2}S}\right)$$

Taking the logarithm of both sides of Equation (9) produces:

(10) $${lnt}_{ind}=ln\frac{K}{J_{n}}+\frac{B}{{ln}^{2}S}$$

The slope *B* can be obtained from plotting ${lnt}_{ind}$ and ${ln}^{2}S$. Then, the data of the interface energy of the crystal can be obtained. The critical nucleation particle size of nucleation kinetics parameters can be obtained from the interface energy data combined with the classical nucleation theory [33].

(11) $$r_{crit}=\frac{2\gamma V_{m}}{k_{B}TlnS}$$

The relationship between growth rate and supersaturation ratio can be expressed as [34]:

(12) $$G=K_{G}f\left(S\right)$$

where $K_{G}$ is a constant of the growth rate independent of the supersaturation ratio and $f\left(S\right)$ depends on the growth mechanism, related to the supersaturation ratio. The expressions of $f\left(S\right)$ are shown in Table 8.

Combining Equations (7), (8) and (12), we obtain:

(13) $$t_{ind}=A_{u}{\left[f\left(S\right)\right]}^{\left(\frac{1}{n}-1\right)}exp\left(\frac{B}{n{ln}^{2}S}\right)$$

where

(14) $$A_{u}={\left(\frac{\alpha }{k_{v}J_{n}{K_{G}}^{n-1}}\right)}^{\frac{1}{n}}$$

For normal growth, spiral growth and diffusion-controlled growth, Formula (12) can be rearranged as:

(15) $$F_{u}\left(S\right)=lnA_{u}+\frac{B}{n{ln}^{2}S}$$

where

(16) $$F_{u}\left(S\right)=ln\left\{t_{ind}{\left[f\left(S\right)\right]}^{\frac{n-1}{n}}\right\}$$

Therefore, it can be seen from Equation (15) that for different *n*, the relationship between $F_{u}\left(S\right)$ and $1/{ln}^{2}S$ is linear.

For two-dimensional nucleation-mediated growth, Equation (14) can be converted to:

(17) $$F_{u}\left(S\right)=lnA_{u}+\left(n-1\right)\frac{B_{2D}}{3nlnS}+\frac{B}{n{ln}^{2}S}$$

where

(18) $$F_{u}\left(S\right)=ln\left\{t_{ind}{{\left(S-1\right)}^{\frac{2\left(n-1\right)}{3n}}S}^{\frac{n-1}{3}}\right\}$$

In this case, a parabolic model is used to fit the relationship between $F_{u}\left(S\right)$ and 1/lnS for different *n*. Depending on the result of fitting, the growth mechanisms were identified as normal growth, spiral growth, volume diffusion-controlled growth or 2D nucleation-mediated growth.

## 5. Conclusions

In this paper, the nucleation induction periods of KCl in the presence of different flotation agents were measured. Through the analysis of induction period data, it was found that there is a good linear relationship between ${lnt}_{ind}$ and ${ln}^{2}S$. The linear relationship consists of two straight lines with different slopes. The straight line with higher slopes indicates that homogeneous nucleation occurs at a higher supersaturation, while the straight lines with low slope indicate that heterogeneous nucleation occurs under low supersaturation. By fitting the interfacial energy data, it is indicated that the flotation agents promoted the homogeneous nucleation of KCl and inhibited the heterogeneous nucleation of KCl. By fitting the experimental data, it was found that the presence of flotation agents increases the nucleation order *m*, and *m*(ODA) > *m*(SDS) > *m*(DAH) > *m*(H_2_O). In addition, different growth mechanisms were used to fit the data of the induction period, and it was shown that the growth of potassium chloride was consistent with the 2D nucleation-mediated growth mechanism, and the addition of flotation agents did not change the growth mechanism of potassium chloride. Finally, the effects of different flotation agents on the crystal habit and particle size distribution of potassium chloride under certain experimental conditions were investigated. Compared with the cooling crystallization in pure water, the particle size of potassium chloride decreased after flotation agent was added, and the particle size increased in the order of ODA < SDS < DAH. The results of particle size distribution are exactly consistent with the order of nucleation order *m*. The larger the nucleation order *m*, the larger the nucleation rate and the smaller the particle size. Therefore, for the production process of KCl, in order to increase the particle size of KCl products, it is suggested to reduce the circulation amount of mother liquor of fine potassium in the selective decomposition crystallization stage or replace it with saturated KCl solution. At the same time, if the mother liquor of fine potassium reflux is used, it is recommended to use cationic surfactants with relatively low amounts of carbon chains as flotation agents on the grounds of ensuring the flotation effect.

## Figures and Tables

**Table 1:** Empirical parameters obtained from the Formula (2) in the presence of different flotation agents.

Flotation Agents	$K_{1}$	δ	*R* ^2^
H_2_O	651.22	20.38	0.9984
ODA	1497.26	31.53	0.9470
DAH	1038.16	27.02	0.9516
SDS	1010.14	26.23	0.9765

**Table 2:** The slopes of the straight lines and the corresponding interfacial energies fitted according to Equation (10).

Flotation Agents	Homogeneous Nucleation					Heterogeneous Nucleation				
	Slope	Intercept	R^2^	γ/mJ·m^−2^	$\frac{J_{n}}{K}$/s^−1^	Slope	Intercept	R^2^	γ/mJ·m^−2^	$\frac{J_{n}}{K}$/s^−1^
H_2_O	0.0087	3.4859	0.9740	5.3934	3.06 × 10^−2^	0.0012	4.9105	0.9570	2.8054	7.37 × 10^−3^
ODA	0.0078	3.6395	0.9964	5.1949	2.63 × 10^−2^	0.0026	4.5085	0.9921	3.6004	1.10 × 10^−2^
DAH	0.0091	3.5037	0.9927	5.1604	3.01 × 10^−2^	0.0024	4.4828	0.9864	3.4860	1.13 × 10^−2^
SDS	0.0076	3.6084	0.9826	5.1434	2.71 × 10^−2^	0.0019	4.7179	0.9909	3.2599	8.93 × 10^−3^

**Table 3:** The critical nucleation sizes *r_crit_* (Å) calculated according to Equation (11).

	Homogeneous Nucleation				Heterogeneous Nucleation			
*S*	H_2_O	ODA	DAH	SDS	H_2_O	ODA	DAH	SDS
1.04	41.62	40.09	39.82	39.69	21.65	27.78	26.90	25.16
1.05	33.46	32.22	32.01	31.91	17.40	22.33	21.62	20.22
1.06	28.01	26.98	26.80	26.72	14.57	18.70	18.11	16.93
1.07	24.13	23.24	23.08	23.01	12.55	16.11	15.59	14.58
1.08	21.21	20.43	20.29	20.23	11.03	14.16	13.71	12.82
1.09	18.94	18.24	18.12	18.06	9.85	12.64	12.24	11.45
1.10	17.13	16.50	16.39	16.33	8.91	11.43	11.07	10.35
1.11	15.64	15.07	14.97	14.92	8.14	10.44	10.11	9.45
1.12	14.40	13.87	13.78	13.74	7.49	9.62	9.31	8.71
1.14	12.46	12.00	11.92	11.88	6.48	8.32	8.05	7.53

**Table 4:** Expressions of *F_u_*(*S*) corresponding to different growth mechanisms.

Growth Mechanism	*ν*	*n*	*F_u_*(*S*)
Normal growth	1	4	$ln\left\{t_{ind}S^{\frac{1}{4}}{\left(S-1\right)}^{\frac{3}{4}}\right\}$
Spiral growth	1	4	$ln\left\{t_{ind}S^{\frac{1}{4}}{\left(S-1\right)}^{\frac{3}{2}}\right\}$
Diffusion-controlled growth	1/2	5/2	$ln\left\{t_{ind}S^{\frac{2}{5}}{\left(S-1\right)}^{\frac{3}{5}}\right\}$
2D nucleation-mediated growth	1	4	$ln\left\{t_{ind}S^{\frac{1}{2}}{\left(S-1\right)}^{\frac{1}{2}}\right\}$

**Table 5:** The coefficients R^2^ for different growth mechanisms of KCl in the presence of different flotation agents.

Flotation Agents	Growth Mechanisms	R^2^	
		(1)	(2)
H_2_O	Normal growth	0.8493	0.9714
	Spiral growth	0.9898	0.8530
	Diffusion-controlled growth	0.9130	0.9729
ODA	Normal growth	0.9963	0.9587
	Spiral growth	0.8217	0.3583
	Diffusion-controlled growth	0.9966	0.9775
DAH	Normal growth	0.9951	0.9978
	Spiral growth	0.6856	0.3683
	Diffusion-controlled growth	0.9938	0.9970
SDS	Normal growth	0.9960	0.9756
	Spiral growth	0.0301	0.0033
	Diffusion-controlled growth	0.9957	0.9798

**Table 6:** The particle size statistics of potassium chloride in the presence of different flotation agents obtained via particle size analyzer.

Flotation Agents	Mean [µm]	D10 [µm]	D50 [µm]	D90 [µm]
H_2_O	357.46	221.45	328.43	549.62
ODA	284.17	166.54	263.26	437.21
DAH	291.5	118.42	283.12	479.18
SDS	289.49	115.30	290.29	460.87

**Table 7:** Different concentrations of flotation agents selected in the experiment.

Flotation Agents	Concentration (mol/L)		
ODA	2 × 10^−6^	8 × 10^−6^	2 × 10^−5^
DAH	2 × 10^−6^	8 × 10^−6^	2 × 10^−5^
SDS	4 × 10^−6^	2 × 10^−5^	1 × 10^−4^

**Table 8:** The $f\left(S\right)$ corresponding to different growth mechanisms.

Growth Mechanism	*f*(*S*)
Normal growth	(*S* − 1)
Spiral growth	(*S* − 1)^2^
Diffusion-controlled growth	(*S* − 1)
2D nucleation-mediated growth	${\left(S-1\right)}^{\frac{2}{3}}S^{\frac{1}{3}}exp\left(\frac{-B_{2D}}{3lnS}\right)$

## Data Availability

The data presented in this study are available on request from the State Key Laboratory of Chemical Engineering, School of Chemical Engineering and Technology, Tianjin University.
